# Hydration Energies of Protonated and Sodiated Thiouracils

**DOI:** 10.1007/s13361-014-0987-8

**Published:** 2014-10-01

**Authors:** Henryk Wincel

**Affiliations:** Institute of Physical Chemistry, Polish Academy of Sciences, 01-224 Warsaw, Poland

**Keywords:** Hydration energies, Protonated and sodiated thiouracils, High-pressure mass spectrometry

## Abstract

Hydration reactions of protonated and sodiated thiouracils (2-thiouracil, 6-methyl-2-thiouracil, and 4-thiouracil) generated by electrospray ionization have been studied in a gas phase at 10 mbar using a pulsed ion-beam high-pressure mass spectrometer. The thermochemical data, *ΔH* ^*o*^ *n*, *ΔS* ^*o*^ *n*, and *ΔG* ^*o*^ *n*, for the hydrated systems were obtained by equilibrium measurements. The water binding energies of protonated thiouracils, [2SU]H^+^ and [6Me2SU]H^+^, were found to be of the order of 51 kJ/mol for the first, and 46 kJ/mol for the second water molecule. For [4SU]H^+^, these values are 3–4 kJ/mol lower. For sodiated complexes, these energies are similar for all studied systems, and varied between 62 and 68 kJ/mol for the first and between 48 and 51 kJ/mol for the second water molecule. The structural aspects of the precursors for hydrated complexes are discussed in conjunction with available literature data.

**Graphical Abstract:**
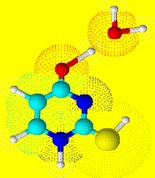
ᅟ

ᅟ

## Introduction

Although thiouracils have been identified as minor components of t-RNA, they are the subject of considerable interest because of their biological and pharmacologic activities.

2-Thiouracil and 4-thiouracil have found medical applications as anticancer and antithyroid drugs [1, 2]. Also 6-methyl-2-thiouracil exhibits antithyroid activity [3]. But these compounds, like other nucleobases, can exist in many tautomeric forms, which may cause base-pairing mismatch leading to mutation during DNA duplication [4].

Information about different tautomeric forms of neutral thiouracils has been inferred from a number of experimental and theoretical studies [5–10], which show that 2-thiouracil, 4-thiouracil, and 2,4-dithiouracil exist as oxo-thione tautomers not only in the gas phase but also in solution and in the solid state. Because of the important role that proton transfer and metal binding to nucleobases can play in their tautomerization [11–14], much effort has gone into investigating the gas-phase proton and alkali metal ion affinities of thiouracils [8, 15–18], and their interaction with cations such, as Ca^2+^ [19], Cu^+^ [20], Cu^2+^ [21, 22], Cd^2+^ [23], or Pb^2+^ [24]. Lamsabhi et al. [15] measured the gas-phase proton affinities of 2-thiouracil, 4-thiouracil, and 2,4-dithiourcil by Fourier transform ion cyclotron resonance mass spectrometry, and calculated the stabilities of their tautomeric forms. These investigations reveal that in all cases, protonation takes place at the heteroatom attached to position 4, and for 2-thiouracil and 2,4-thiouracil, the most stable protonated conformer is the enol-enethiol form.

Yang and Rodgers [18] studied extensively the interactions of proton and alkali metal ions (Li^+^, Na^+^ and K^+^) with uracil [U] and five thiourcils (2-thiouracil [2SU], 5-methyl-2-thiouracil [5Me2SU], 6-methyl-2-thiouracil [6Me2SU], 4-thiouracil [4SU], and 2,4-dithiouracil [24dSU]) by guided ion beam mass spectrometry, where the alkali metal–thiouracil complexes were formed by three-body associative reactions and collisionally stabilized in a flow tube ion source. These experiments and theoretical calculations showed that thioketo substitution leads to an increase in the stability of nucleic acids by increasing the proton affinity and acidity of uracil. The effect of thioketo substitution on the alkali metal ion binding affinity and the stability of base pairing are dependent upon the position of substitution. 2-Thioketo substitution leads to an increase in both the alkali metal ion binding affinity and the base pairing energy, whereas 4-thioketo substitution leads to a decrease in both. Results of these studies also show that the alkali metal cation binding to uracil and thiouracils decreases monotonically with increasing size of the metal cation, confirming the electrostatic nature of the binding.

Rodgers and co-workers [25, 26] recently also provided direct experimental characterization of the structures of the protonated and sodiated complexes of uracil and thiouracils mentioned above using infrared multiple photon dissociation (IRMPD) action spectroscopy in conjunction with electronic structure calculations. These complexes were generated by electrospray. Results [25] for the ground-state structures of [U]H^+^, [2SU]H^+^, [5Me2SU]H^+^, [5Me2SU]H^+^, [6Me2SU]H^+^, and [24dSU]H^+^ show that protonation leads to preferential stabilization of a minor tautomer of the nucleobase, where both keto (thioketo) groups are converted to hydroxyl (sulfhydryl) groups by proton binding and proton transfer from the neighboring N3H group, whereas the ground-state structure for [4SU]H^+^ corresponds to protonation of the canonical keto-thioketo tautomer at the 4-thioketo position. For the [U]H^+^ and [4SU]H^+^ complexes, evidence for the presence of low-energy conformers in very low abundance was presented. Similar studies [26] on sodiated complexes indicate that in the case of the ground-state structures of [U]Na^+^, [2SU]Na^+^, [5Me2SU]Na^+^, and [6Me2SU]Na^+^, the sodium cation preferentially binds to the 4-keto group of the canonical 2,4-diketo or 2-tioketo-4-keto tautomer in these complexes. In contrast, binding of a sodium cation in [4SU]Na^+^ and [24dSU]Na^+^ preferentially stabilizes an alternative 2-keto-4-sulfhydryl or 2-tioketo-4-sulfhydryl tautomer.

Most biological processes occur in an aqueous environment, and hydration plays a very important role in biological systems. Water is vital for the stability of the double helix of DNA [27, 28], whereas metal ions like Na^+^, K^+^, and Ca^2+^ are essential for charge compensation of the negatively charged phosphate sugar backbone and play important roles in biological functions, such as the regulation of enzymes and stabilization of nucleic acids, and are responsible for osmotic equilibrium in cells [29–36]. Water and ions form more long-lived pairs with the sites on DNA bases than near phosphate groups [31]. This has an implication for the study of the interactions of individual bases with metal ions and water molecules as well as hydration of the complexes. The effect of hydration can be examined on a microscopic level by investigating the interactions of neutral and/or ionic biological systems with a few water molecules. Kryachko et al. [16, 17] theoretically investigated the interaction of a water molecule with the neutral, protonated, and deprotonated forms of 2-thiouracil, 4-thiouracil, and 2,4-dithiourcil, and determined the water binding energies in the hydrated complexes. Bakker et al. [37] studied the singly hydrated protonated uracil by IRMPD spectroscopy, and they show that the two protonated keto and enolic forms of uracil directly originate from electrospray, and the protonated enolic tautomers and their hydrated structures are predominant. Similarly, Fridgen and co-workers [38] used IRMPD spectroscopy in combination with theoretical calculations to determine the structures of the hydrated complexes of lithium-cationized uracil and thymine, BLi^+^(H_2_O)_*n* = 1,2_ (B = uracil, thymine). They show that the lithium cation in all complexes is bound to the O4 oxygen of the base, and the first two water molecules are coordinated to Li^+^.

**Scheme 1:**
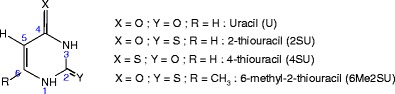
Atom numbering in uracil and the thiouracils

In the present paper, we report the first experimental results on the hydration of protonated and sodiated thiouracils: 2-thiouracil, 4-thiouracil and 6-methyl-2-thiouracil Scheme 1

.

This work is a continuation of previous studies [39, 40] on the gas-phase microhydration of protonated and alkali metal (Na^+^ and K^+^) cationized nucleic acid bases generated by electrospray ionization (ESI). 

## Experimental

The gas-phase hydration experiments were performed with a home-made 60° magnetic sector high-pressure mass spectrometer using a pulsed ion-beam ESI ion source, which has been previously described in detail [41]. Briefly, the protonated thiouracils were produced by the electrospray of solutions containing ~2.0 mM nucleobase in a water/methanol (1:1) mixture. The sodiated thiouracils were generated by electrospraying water/methanol (1:1) solutions containing ~2.0 mM nucleobase and NaCl. Each solution was supplied to a silica capillary (15 μm i.d., 150 μm o.d.) by a syringe pump at a rate of 0.8 μL/min. As in the case of the work in [26], the intensities of sodium cationized thiouracils formed by ESI dramatically decreased in the order [6Me2SU]Na^+^>[2SU]Na^+^>[4SU]Na^+^. The following nucleobase samples were used: 2-thiouracil (98% Alfa Aesar GmbH & Co. KG, Karlsruhe, Germany), 4-thiouracil (97% Acros Organics, New Jersey, USA), and 6-methyl-2-thiouracil (98% abcr GmbH & Co.KG, Karlsruhe, Germany). The clustered ions were desolvated by a dry nitrogen gas counter-current and in a heated (~80°C) pressure-reducing capillary through which they were introduced into the fore-chamber, and then deflected toward a 3-mm orifice in the interface plate leading to the reaction chamber (RC). Ions drifting across the RC toward the exit slit under the influence of a weak electric field (2 V/cm at 10 mbar) were hydrated and reached equilibrium prior to being sampled to the mass analysis section of the mass spectrometer. Ion detection was provided by a secondary electron scintillation detector of the Daly type with an aluminum conversion dynode using a short rise-time photomultiplier (Type R-647-04, Hamamatsu Photonics, Deutschland GmbH, Germany). The output pulses of the multiplier were counted using a multichannel scaler with dwell-time per channel of 1 μs.

**Figure 1:**
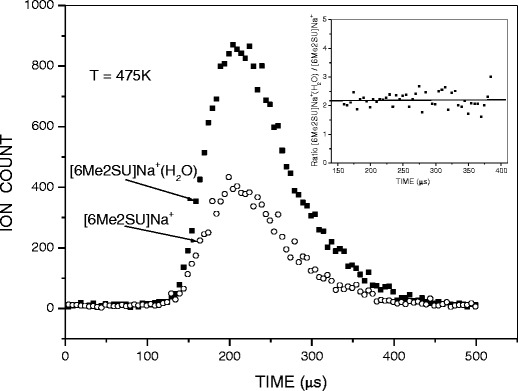
Arrival time distributions of the reactant, [6Me2SU]Na^+^, and product, [6Me2SU]Na^+^ ٠(H_2_O), ions. The inset shows the ratio of ion intensities, [6Me2SU]Na^+^(H_2_O)/[6Me2SU]Na^+^ as a function of ion residence time

Mass spectra were registered with continuous ion sampling, while for equilibrium determination the ion beam was injected into the RC in a pulsed mode by applying short pulses (+52 V, 90–200 μs) to the deflection electrode. Typically, several thousand injection pulses were sufficient to accumulate a reasonable signal of the ion arrival time distribution (ATD) for each mass on the multichannel scaler (Fig. 1).

The reagent gas mixture consisting of pure N_2_ as the carrier gas at about 10 mbar and a known partial pressure of water vapor (0.02–0.20 mbar) was supplied to the RC via the heated reactant gas inlet (RGI) at a flow rate of ~100 mL/min. The pressure was measured with an MKS capacitance manometer attached near the inlet of the RGI. The amount of water introduced into the N_2_ gas flow was kept constant throughout the temperature-dependent measurements of the equilibrium constants. Water concentrations were controlled continuously with a calibrated temperature and humidity transmitter (Delta OHM, Type DO 9861 T; Italy) inserted into the carrier gas flow line. The RC temperature was monitored by an iron-constantan thermocouple, which was embedded close to the ion exit slit; the temperature could be varied from ambient to 300°C by electrical heaters.

The equilibria studied for the hydration of protonated and sodiated thiouracils can be described by the general reaction 1

1 $$ \left[\mathrm{S}\mathrm{U}\right]{\mathrm{A}}^{+}\cdot {\left({\mathrm{H}}_2\mathrm{O}\right)}_{n-1}+{\mathrm{H}}_2\mathrm{O}\leftrightarrow \left[\mathrm{S}\mathrm{U}\right]{\mathrm{A}}^{+}\cdot {\left({\mathrm{H}}_2\mathrm{O}\right)}_n\mathrm{A}=\mathrm{H}\mathrm{a}\mathrm{nd}\mathrm{N}\mathrm{a}\mathrm{S}\mathrm{U}=2\mathrm{S}\mathrm{U},4\mathrm{S}\mathrm{U}\mathrm{a}\mathrm{nd}6\mathrm{Me}2\mathrm{S}\mathrm{U} $$

for which the thermodynamic equilibrium constant is

2 $$ {K}_{n-1,n}=\left({I}_n\cdot {P}_o/{I}_{n-1}\cdot P\right) $$

where *I*_*n*_ and *I*_*n*-1_ are recorded ATD peak areas of [SU]A^+^(H_2_O )_*n*_ and [SU]A^+^(H_2_O )_*n*-1_ , respectively, and *P* is the known partial pressure of water (in mbar).The standard pressure *P*_*o*_ is 1000 mbar. Equilibrium attainment in the RC was verified by comparing the ATDs of the reactant and product ions, and testing that the *I*_*n*_ /*I*_*n*-1_ ratio was independent of ion residence time. A typical example of such tests is shown in Fig. 1 for the (0,1) hydration step of 6Me2SUNa^+^. The inset of the figure shows that within the error limits and the limits of statistical noise, the ratio {[6Me2SU]Na^+^(H_2_O)}/{[6Me2SU]Na^+^} remains essentially constant, suggesting the attainment of equilibrium for the system.

Measuring *K*_*n*-1,*n*_ as a function of temperature *T* and using the thermodynamic relationships 3 and 4

3 $$ \ln {K}_{n- 1,n}=\left(\varDelta {S^o}_n/R\right)-\left(\varDelta {H^o}_n/RT\right) $$

4 $$ \varDelta {G^o}_n=\varDelta {H^o}_n-T\varDelta {S^o}_n $$

the values for the enthalpy, *ΔH* ^*o*^_*n*_, entropy, *ΔS* ^*o*^_*n*_, and free energy, *ΔG* ^*o*^_*n*_, of reaction 1 were obtained. The weighted least-squares fitting procedure was used to obtain the slopes and intercepts of each line. The slopes determine the enthalpy change (–*ΔH* ^*o*^_*n*_) and the intercepts yield the corresponding *ΔS* ^*o*^_*n*_ value. The uncertainty corresponds to the standard deviation of the linear least-squires fit. Only data for small *n* are given in the tables, because determining the enthalpies for higher hydration steps requires equilibration temperatures below room temperature, which is not possible with the present reaction chamber.

During these experiments, we measured the hydration energies for reaction 5 to support the validity of the present results and provide bases for comparison with the data obtained in previous studies.

5 $$ {\left({\mathrm{CH}}_3\right)}_2{{\mathrm{NH}}_2}^{+}\cdot \left({\mathrm{H}}_2\mathrm{O}\right)+{\mathrm{H}}_2\mathrm{O}\leftrightarrow {\left({\mathrm{CH}}_3\right)}_2{{\mathrm{NH}}_2}^{+}\cdot {\left({\mathrm{H}}_2\mathrm{O}\right)}_2 $$

Table 1 shows that the values measured in this work are in good agreement with those measured previously [41–43].

**Table 1:** Experimental Enthalpies, Entropies, and Free Energy Values^a^ for the Hydration of Protonated Systems

Ion	*n*	–*ΔH* ^*o*^_*n*_ (kJ/mol)	–*ΔS* ^*o*^_*n*_ (J/mol K)	–*ΔG* ^*o*^_*n*_ (kJ/mol)^b^	PA (kJ/mol)
[U]H^+^	1	51.9(2)^c^	66.1(5)^c^	32.2(4)^c^	879.5^d^; 872.7^e^; 870.3^f^
	2	46.9(2)^c^	77.0(6)^c^	23.9(5)^c^	
[2SU]H^+^	1	51.0(3)	80.3(8)	27.1(5)	879.5^d^; 879.5^g^; 874.9^f^
	2	46.0(2)	80.7(8)	22.0(5)	
[4SU]H^+^	1	46.9(2)	74.9(3)	24.6(3)	888.9^d^; 882.8^g^;
	2	41.8(1)	81.2(3)	17.6(2)	
[6Me2SU]H^+^	1	50.6(1)	89.5(2)	23.6(2)	895.7^d^
	2	46.9(2)	98.7(7)	17.5(4)	
(CH_3_)_2_NH_2_^+^	2	56.5(3)	109.6(9)	23.8(6)	
		55.6(4)^h^	110.0(10)^h^	22.8(7)^h^	
		57.7(3)^i^	112.1(10)^i^	24.3(6)^i^	
		56.5(2)^j^	103.3(4)^j^	25.5(3)^j^	

Standard pressure is 1000 mbar.

^a^ Uncertainties in parentheses.

^b^ –*ΔG* ^*o*^_*n*_ at 298 K.

^c^ Reference [39].

^d^ Calculated values at the MP2(full)/6-311+G(2d,2p) level of theory using MP2(full)/6-31G* optimized geometries, Reference [18].

^e^ Exp. from Reference [44].

^f^ Exp. from Reference [8].

^g^ Exp. from Reference [15].

^h^ Reference [41].

^I^ Reference [43].

^j^ Reference [42].

## Results and Discussion

### Hydration of Protonated Thiouracils

**Figure 2:**
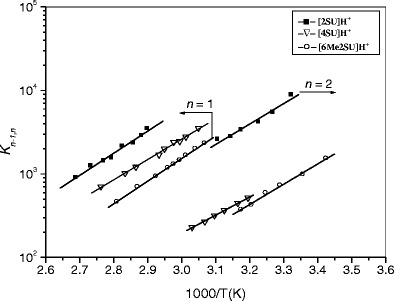
van’t Hoff plots of equilibrium constants for the gas-phase reactions [SU]H^+^·(H_2_O)_*n*-1_ + H_2_O <=> [SU]H^+^·(H_2_O)_*n*_

The van’t Hoff plots for the hydration reactions of protonated 2SU, 4SU, and 6Me2SU are shown in Fig. 2, and the thermochemical data obtained from these van’t Hoff plots are summarized in Table 1, along with the reported values for the protonated uracil [39] for comparison. These results show that the hydration enthalpies (–*ΔH* ^*o*^_*n*_) of [2SU]H^+^ and [6Me2SU]H^+^ are very similar to that of [U]H^+^, while being slightly lower for [4SU]H^+^ than for those ions. These observations may be understood as the result of conformational differences in the protonated forms of the nucleobases as the precursors for the hydrated systems. As mentioned in the Introduction section, the experimental IRMPD and computational studies by Rodgers et al. [25, 26] have provided very useful information about the structures of protonated and sodium-cationized uracil and thiouracils formed by ESI. Their results show that for [2SU]H^+^ and [6Me2SU]H^+^, as in the case of [U]H^+^, the ground-state enolic form, **1**, is the dominant conformer.

The first excited-state conformers, **1a**, of [2SU]H^+^ and [6Me2SU]H^+^, were found to be 6.8 and 7.4 kJ/mol higher in Gibbs energy than **1**, respectively, and would be expected to be small abundance structures [25]. It is, therefore, reasonable to assume that similar populations of the ground and first excited states of [2SU]H^+^ and [6Me2SU]H^+^ are produced by ESI in the present experiments, and these two conformers, **1** and **1a**, are the precursors for hydrated microclusters. For the conformer **1**, the positive Mulliken charges predicted [25] by the B3LYP/6-311+ G(2d,2p) calculations for the hydrogen atoms of the O4–H and S2–H bonds are equal to 0.316 and 0.128e in [2SU]H^+^ and 0.311 and 0.125e in [6Me2SU]H^+^, respectively (unpublished results from [25] and [26], personal communication from M.T. Rodgers).

**Scheme 2:**
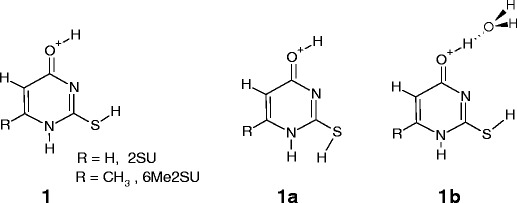
Schematic diagram of the ground-state (**1**) and the first-excited state (**1a**) conformers of protonated 2-thiouracil and 6-mehyl-2-thiouracil, and the hydrated complex of **1**

This suggests that the O4H position could be the favorable site for a water molecule binding, **1b**, as schematically depicted in Scheme 2.

In the case of [U]H^+^, the Mulliken charges on the H atoms at the O2H and O4H positions are predicted, [25] to be 0.342 and 0.317e respectively, and these positions are the preferred binding sites for the H_2_O attachment. This fact is reflected in the formation of singly hydrated protonated structures resulting from the H_2_O addition to both sites, O2H and O4H, of the ground-state structure of [U]H^+^, [37]. The first excited-state conformer of [U]H^+^, where the proton at the O4H position is directed away from the adjacent N3H group of the canonical diketo form, is higher in energy by 5.6 kJ/mol than the ground-state conformer **1**, and would be expected to be a minor abundance [25].

**Scheme 3:**
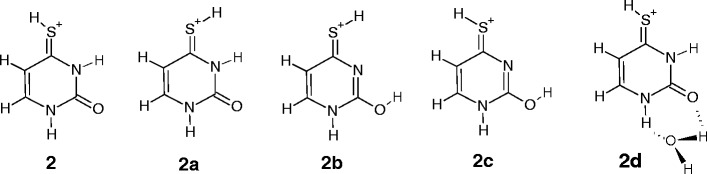
Structures of protonated 4-thiouracil considered in this study

For the [4SU]H^+^ complex, the theoretical study by Lamsabhi et al. [15] and the experimental IRMPD/computation studies by Rodgers et al. [25] clearly indicate that the ground-state structure corresponds to the canonical keto-thioketo tautomer, **2**, and other tautomeric forms, **2a**, **2b**, and **2c**, are less stable than **2** by 1.6, 3.0, and 6.6 kJ/mol, respectively. The predicted IR spectra for **2** and **2a** are nearly the same, and these structures cannot be distinguished spectroscopically [25]. Because the barrier for **2** → **2a** conversion is very small (23.0 kJ/mol [15], 30.2 kJ/mol [25]), it is most likely that the **2a** conformer substantially contributes to the ion population of [4SU]H^+^ generated by ESI. On the other hand, for the **2** → **2b** and **2** → **2c** conversions, the energy barriers are considerably higher than those for **2** → **2a** (165 kJ/mol and 169 kJ/mol, respectively [15]), and the **2b** and **2c** conformers (if formed) would be expected to be present in a small abundance Scheme 3.

It is, therefore, very likely that under the present experiment, the **2** and **2a** conformers should be the dominant precursors for the hydrated complexes, [4SU]H^+•^(H_2_O)_*n* = 1,2_. The positive charge (110e [25]) on the H atom of the S4–H bond in **2** is much smaller compared with that of O4–H in [2SU]H^+^, (0.316e). Therefore, the suggestion of reactive site for water interaction with S4H of **2** seems to be very problematic. Based on the calculations at the B3LYP/6-31+G(d,p) level of theory, Kryachko et al. [17] predicted that the positive Mulliken net charges at the N1H and N3H positions in the structure **2** are equal to 0.376e and 0.371e , respectively, and the interaction between this structure and one water molecule leads to the **2d** complex, where the N1H and C2O sites of **2** form the hydrogen bonds with H_2_O. The calculated [17] binding energy of water in this complex (61.7 kJ/mol) is significantly higher than that measured in the present experiments (46.9 kJ/mol).

**Figure 3:**
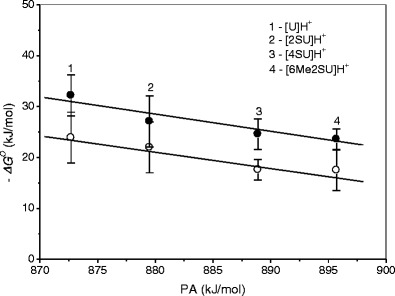
Correlation between hydration free energy, –*ΔG* ^*o*^, at 298 K and corresponding proton affinity, PA, of uracil and thiouracils for the first (●) and second (○) water molecule. For 2SU, 4SU, and 6Me2SU, the PA values are taken from Reference [18]; for U from Reference [44]; see Table 1

The reaction enthalpies (–*ΔH* ^*o*^_*n* = 1,2_) for the hydration of the [2SU]H^+^ and [6Me2SU]H^+^ complexes are very similar to that of [U]H^+^, whereas for [4SU]H^+^ is slightly lower (Table 1). The free energy values (–*ΔG* ^*o*^_*n* = 1,2_ ) for the hydration of these complexes follow the order [U]H^+^>[2SU]H^+^>[4SU]H^+^>[6Me2SU]H^+^. Although the differences between these values are small and generally fall within the combined experimental uncertainty, the general trend is evident. The hydration free energy decreases as the proton affinity (PA) of the nucleobase increases (Fig. 3). This trend is consistent with the correlation between the binding energies of hydrogen-bonded complexes [B]H^+…^OH_2,_ where B is an organic base, and the proton affinity difference ΔPA=PA(B) – PA(H_2_O) [45]. This results from the fact that when the PA of neutral B increases, the residual charge on the proton in the BH^+…^OH_2_ complex becomes smaller, resulting in a weaker electrostatic interaction of H_2_O with [B]H^+^. Such a correlation has been experimentally observed for many protonated systems [39, 45–49]. The lower values of –*ΔG* ^*o*^_2_ compared with –*ΔG* ^*o*^_1_ can be attributed to the charge delocalization on the binding site with the addition of the second water molecule.

The effect of thioketo and thioketo plus methyl substitution on the free energy changes of [U]H^+^(H_2_O)_*n* = 1,2_ can be deduced from the data given in Table 1. For [2SU]H^+^(H_2_O)_*n* = 1,2,_ the decrease in the –*ΔG* ^*o*^_*n* = 1,2_ values upon 2-thioketo substitution is ~5.1 kJ/mol for the first H_2_O and ~2 kJ/mol for the second one. Methylation of [2SU]H^+^(H_2_O)_*n* = 1,2_ leads to a further decrease in the –*ΔG* ^*o*^_*n* = 1,2_ values by ~9 kJ/mol for *n* = 1 and ~6 kJ/mol for *n* = 2. In the [4SU]H^+^(H_2_O)_*n* = 1,2_ clusters_,_ 4-thioketo substitution results in decrease in the *ΔG* ^*o*^_*n* = 1,2_ values by ~8 kJ/mol for *n* = 1 and ~6 kJ/mol for *n* = 2.

**Figure 4:**
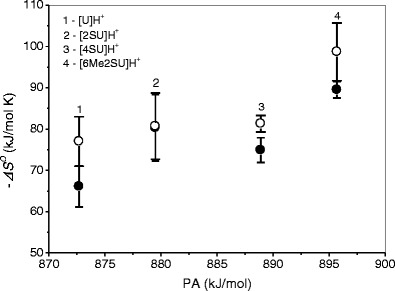
Plot of hydration entropy, –*ΔS* ^*o*^, versus corresponding proton affinity, PA, of uracil and thiouracils for the first (●) and second (○) water molecule. For 2U, 4SU, and 6Me2SU, the PA values are taken from Reference [18]; for U from Reference [44]; see Table 1

The influence of thioketo and methyl substitution on the entropies of hydration of uracil is reflected in the –*ΔS* ^*o*^_*n* = 1,2_ values plotted as function of PA in Fig. 4 and summarized in Table 1. These data show that with increasing PA, the –*ΔS* ^*o*^_*n* = 1,2_ values increase. This implies that by forming the stronger proton binding to the thiouracils, the H_2_O ligands in the [SU]H^+^(H_2_O)_*n* = 1,2_ clusters lose in the freedom of motion. The increase in –*ΔS* ^*o*^_*n*_ with *n* = 1– > 2 observed for these clusters may be attributed to an increase in the restriction of motion for ligands due to the exchange repulsion at *n* = 2. The 2-thioketo substitution results in an increase in the –*ΔS* ^*o*^_*n* = 1,2_ values by ~14 (*n* = 1) and ~3 (*n* = 2) J/mol K, and 6-methylation of [2SU]H^+^(H_2_O)_*n* = 1,2_ enhances these values by ~23 (*n* = 1) and ~22 (*n* = 2) J/mol K compared with [U]H^+^(H_2_O)_*n* = 1,2_. The 4-thioketo substitution results in a smaller increase in the –*ΔS* ^*o*^_*n* = 1,2_ values for *n* = 1 (~9 J/mol K) and somewhat larger for *n* = 2 (~4 J/mol K) compared with [2SU]H^+^(H_2_O)_*n* = 1,2_.

### Hydration of Sodiated Thiouracils

**Figure 5:**
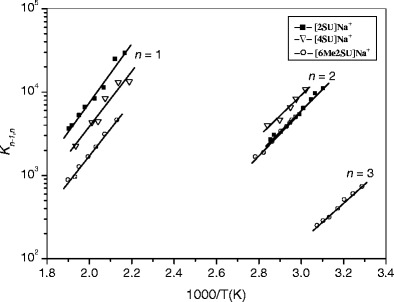
van’t Hoff plots of equilibrium constants for the gas-phase reactions [SU]Na^+^·(H_2_O)_*n*-1_ + H_2_O <=> [SU]Na^+^·(H_2_O)_*n*_

Examples of the van’t Hoff plots used to obtain the thermochemical data (Table 2) for the hydration of sodiated thiouracils are shown in Fig. 5. Unfortunately, it was not possible to determine the enthalpies for further hydration steps of [2SU]Na^+^(H_2_O)_*n* = 1,2_ and [4SU]Na^+^(H_2_O)_*n* = 1,2_ because the intensities of these complexes were too low. Table 2 shows that for all sodium-cationized nucleobases, the hydration enthalpies (–*ΔH* ^*o*^_*n*_) as well as the corresponding (–*ΔG* ^*o*^_*n*_) values are similar within experimental uncertainty.

**Table 2:** Experimental Enthalpies, Entropies, and Free Energy Values^a^ for the Hydration of Sodiated Uracil and Thiouracils

Ion	*n*	–*ΔH* ^*o*^_*n*_ (kcal/mol)	–*ΔS* ^*o*^_*n*_ (cal/mol K)	–*ΔG* ^*o*^_*n*_ (kcal/mol)^b^	MIA (kJ/mol)
[U]Na^+^	1	62.3(2)^c^	66.5(4)^c^	42.6(3)^c^	146.2^d^; 134.6^g^
	2	50.6(2)^c^	70.3(5)^c^	29.7(3)^c^	
[2SU]Na^+^	1	67.8(4)	61.1(8)	49.4(7)	143.5^d^; 139.8^g^
	2	48.9(2)	72.0(5)	27.5(4)	
[4SU]Na^+^	1	63.6(7)	58.6(15)	46.1(11)	138.9^e^; 151.1^f^
	2	47.7(5)	66.5(13)	27.9(9)	
[6Me2SU]Na^+^	1	62.8(3)	62.8(7)	44.1(5)	150.6^d^; 143.6^g^
	2	51.0(2)	80.3(5)	27.1(4)	
	3	44.4(2)	89.5(6)	17.7(4)	

Standard pressure is 1000 mbar.

^a^ Uncertainties in parentheses.

^b^ –*ΔG* ^*o*^_*n*_ at 298 K.

^c^ Reference [40].

^d^ Calculated at the MP2(full)/6-311+G(2d,2p) level of theory using MP2(full)/6-31G* optimized geometries, Reference [18].

^e^ Calculated on MP2(full) single point energies, Reference [26].

^f^ Calculated on B3LYP single point energies, Reference [26].

^g^ Exp. Reference [18].

**Scheme 4:**
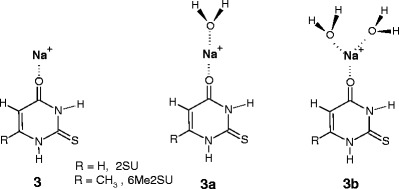
The ground-state conformer of the sodiated 2-thioutacil and 6-methyl-2-thiouracil, and its complexes with water molecule

The experimental IRMPD spectra of [U]Na^+^, [2SU]Na^+^, and [6Me2SU]Na^+^ along with their calculated infrared spectra, reported in the literature [26], show that the ground-state structures for each of these complexes are the conformers **3**, where the sodium cation binds to the O4 carbonyl oxygen atom of their canonical tautomers, as illustrated in Scheme 4.

The first-excited conformers of [2SU]Na^+^ and [6Me2SU]Na^+^ are higher in Gibbs free energy than the ground-state ones by 15.6 and 12.6 kJ/mol, respectively, and would not be expected to be present in the experiments [26]. Based on these observations, one may assume that in the gas phase under our electrospray conditions, the ground-state conformer, **3**, would be the solely existing precursor to the hydrated complexes, **3a** and **3b**.

The Mulliken charges on the sodium cation of the O4^…^Na^+^ bonds in **3** are predicted [26] to be 0.929e for [U]Na^+^ and [2SU]Na^+^, and 0.919e for [6Me2SU]Na^+^. Therefore, it is reasonable to expect that Na^+^ is the most favorable site for water binding in these complexes, **3a** and **3b**. As noted in the Introduction section, Fridgen and co-workers [38] in their IRMPD/computation study found that the Li^+^ cation in the BLi^+^(H_2_O)_*n* = 1,2_ (B = uracil, thymine) complexes is coordinated to the O4 atom, and the first two water molecules of these complexes are attached to the lithium cation.

**Scheme 5:**
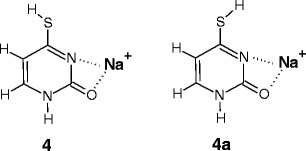
The ground-state (**4**) and the first-excited state (**4a**) conformers of sodiated 4-thiouracil

For the [4SU]Na^+^ complex, the ground-state structure, **4**, generated by ESI was found [26] to be the 2-keto-4-sulfhydryl tautomer of 4SU, where the sulfhydryl hydrogen atom is directed away from the N3 group and Na^+^ is bound to the O2 and N3 atoms, as shown in Scheme 5. The first-excited conformer, **4a**, is 3.9 kJ/mol higher in free energy and the hydrogen atom is oriented toward the N3 atom. The results obtained [26] indicate that these structures are spectroscopically indistinguishable because their calculated IR spectra are virtually the same.

These data also show that other higher energy structures are not present in the experiments, based on the comparison of their calculated spectra with the experimental spectrum [26]. Thus, it would be expected that in our experiments, the conformer **4** alone or both **4** + **4a** are the precursors for [4SU]Na^+^(H_2_O)_*n* = 1,2_. The Mulliken charge predicted [26] for the Na^+^ cation of the **4** structure is equal to 0.746e, and Na^+^ is also the most favorable site for water attachment.

**Figure 6:**
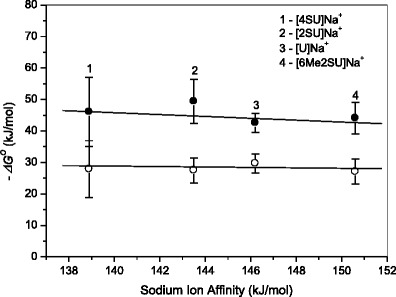
Plot of the binding free energy, –*ΔG* ^*o*^, at 298 K for the first (●) and second (○) water molecule versus corresponding sodium ion affinity of uracil and thiouracils. For U, 2-SU, and 6Me2SU, the calculated sodium ion affinity values are taken from Reference [18]; for 4SU from Reference [26]; see Table 2

As mentioned above, the water-binding energies for the [U]Na^+^ and [SU]Na^+^ complexes are similar within experimental uncertainty (see Fig. 6 and Table 2). This likely reflects the fact that the interaction between Na^+^ and the nucleobase in these complexes is essentially electrostatic [18, 26], and the binding strengths between the complexes and H_2_O is determined by the charge retained on Na^+^ in [U]Na^+^ and [SU]Na^+^, which is expected to decrease as the metal ion affinity (MIA) of the neutral nucleobase increases. Table 2 shows that the MIA values for uracil [50] and thiouracils [18] are all similar, spanning a 12 kJ/mol range. The experimentally derived MIA values are also similar within 7 kJ/mol.

## Conclusions

In this work we examined the hydration of protonated and sodiated thiouracils, [SU]A^+^, where A = H and Na, and SU = 2-thiouracil, 4-thiouracil, and 6-methyl-2thiouracil, using pulsed high-pressure mass spectrometry with electrospray ionization. The structural and energetic aspects of [SU]A^+^ as the precursors for the [SU]A^+^(H_2_O)_*n* = 1,2_ complexes have been discussed based on the experimental IRMPD and computational studies of protonated and sodiated uracil and thiouracils by Rodgers et al. [25, 26]. The obtained hydration enthalpies for [2SU]H^+^ and [6Me2SU]H^+^ are very similar to that of [U]H^+^, while being slightly lower for [4SU]H^+^. These differences may be attributed to a difference in structures of hydrated complexes. For [2SU]H^+^, [6Me2SU]H^+^, and [U]H^+^, the ground-state enolic form is the dominant conformer, whereas for [4SU]H^+^ the ground state corresponds to the canonical keto-thioketo tautomer. The hydration entropies and free energy changes are determined and related to the proton affinities of nucleobases. Thioketo substitution and/or thioketo plus methylation of uracil, by forming the stronger hydrogen binding of protons with uracil, leads to an increase in the hydration entropy, and the H_2_O ligands in the [SU]H^+^(H_2_O)_*n* = 1,2_ complexes lose in the freedom of motion. A correlation between the free energy changes for the addition of the first and second water molecules to [SU]H^+^ and the corresponding proton affinities is observed. Generally, the hydration free energy becomes weaker as the PA increases. For [SU]Na^+^, the hydration enthalpies and the corresponding free energies are similar within experimental uncertainty, and higher than those of [SU]H^+^. For the [U]Na^+^, [2SU]Na^+^, and [6Me2SU]Na^+^ ions, the ground-state structures of the canonical tautomer, in which the sodium cation binds to the O4, are assumed to be the precursors for the hydrated complexes. In the case of [4SU]Na^+^, the most likely candidate for the precursor of hydrated complexes is the 2-keto-4-sulfhyhydryl tautomer. The sodium cation is the most favorable site for water attachment in the [SU]Na^+^(H_2_O)_*n* = 1,2_ systems.
